# Strain effects on the work function of an organic semiconductor

**DOI:** 10.1038/ncomms10270

**Published:** 2016-02-01

**Authors:** Yanfei Wu, Annabel R. Chew, Geoffrey A. Rojas, Gjergji Sini, Greg Haugstad, Alex Belianinov, Sergei V. Kalinin, Hong Li, Chad Risko, Jean-Luc Brédas, Alberto Salleo, C. Daniel Frisbie

**Affiliations:** 1 Department of Chemical Engineering and Materials Science, University of Minnesota, 421 Washington Ave SE, Minneapolis, 55455 Minnesota, USA; 2 Department of Materials Science and Engineering, Stanford University, 476 Lomita Mall, Stanford, 94305 California, USA; 3 Laboratoire de Physico-chimie des Polymères et des Interfaces, Université de Cergy-Pontoise, 5 Mail Gay Lussac, Neuville sur Oise, Cergy-Pontoise Cedex 95031, France; 4 Physical Science and Engineering Division, Solar & Photovoltaics Engineering Research Center, King Abdullah University of Science and Technology (KAUST), Thuwal 23955-6900, Saudi Arabia; 5 Characterization Facility, University of Minnesota, 100 Union St SE, Minneapolis, 55455 Minnesota, USA; 6 Center for Nanophase Materials Sciences, Oak Ridge National Laboratory, 1 Bethel Valley Rd., Oak Ridge, 37831 Tennessee, USA; 7 School of Chemistry and Biochemistry & Center for Organic Photonics and Electronics, Georgia Institute of Technology, Atlanta, 30332 Georgia, USA; 8 Department of Chemistry & Center for Applied Energy Research, University of Kentucky, Lexington, 40506 Kentucky, USA

## Abstract

Establishing fundamental relationships between strain and work function (WF) in organic semiconductors is important not only for understanding electrical properties of organic thin films, which are subject to both intrinsic and extrinsic strains, but also for developing flexible electronic devices. Here we investigate tensile and compressive strain effects on the WF of rubrene single crystals. Mechanical strain induced by thermal expansion mismatch between the substrate and rubrene is quantified by X-ray diffraction. The corresponding WF change is measured by scanning Kelvin probe microscopy. The WF of rubrene increases (decreases) significantly with in-plane tensile (compressive) strain, which agrees qualitatively with density functional theory calculations. An elastic-to-plastic transition, characterized by a steep rise of the WF, occurs at ∼0.05% tensile strain along the rubrene *π*-stacking direction. The results provide the first concrete link between mechanical strain and WF of an organic semiconductor and have important implications for understanding the connection between structural and electronic disorder in soft organic electronic materials.

## Abstract (web summary)

The understanding of strain effect on electronic properties of organic semiconductors is crucial for the designs of flexible electronics. Here, Wu *et al.* characterize the tensile and compressive strain effects on the work function of rubrene single crystals as a benchmark system.

## Graphical abstract

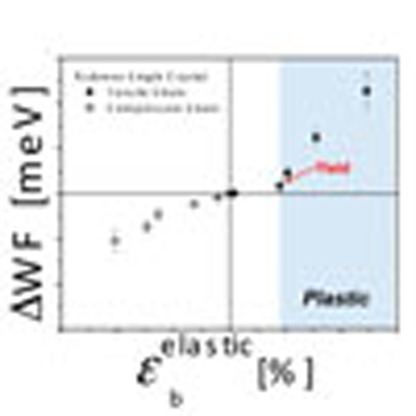

Strain is known to play a critical role in the physical properties of many conventional inorganic semiconductors. For example, strain modifies the band structure and carrier mobilities of group IV materials including Si and Ge, as well as III–V materials such as GaAs^1^,^2^,^3^,^4^,^5^. Consequently, controlled introduction of strain is widely employed to engineer the transport and optical properties of these semiconductors for enhanced device performance^6^,^7^. Similarly, in soft organic semiconductors that serve as the active components in many organic electronic devices^8^,^9^,^10^,^11^,^12^, tensile and compressive strains modify the material electronic properties and function. An intriguing example is the observation by Bao and colleagues that the charge mobility in films of the benchmark organic material 6,13-Bis(triisopropylsilylethynyl)pentacene (TIPS-pentacene) is enhanced under lattice compression^13^. Controlling and understanding strain effects is thus particularly important for device applications of organic semiconductors, especially in the area of flexible electronics, where externally applied strains are routine. Both strain-resistant and strain-sensitive device responses^14^,^15^,^16^,^17^,^18^,^19^ are desirable in flexible circuits, and better knowledge of strain–property relationships will facilitate flexible device designs.

Importantly, better understanding of strain–electrical property relationships is also critical for theoretical models of transport in organic semiconductors. Virtually all organic semiconductor films exhibit intrinsic non-uniform strains arising from lattice or thermal expansion mismatch with the substrate, the presence of defects or post deposition treatments such as thermal annealing^20^,^21^,^22^,^23^. Non-uniform strains in turn lead to variations in intermolecular electronic coupling and thus to local differences in bandwidths and band gaps^24^,^25^, that is, ‘raggedy' valence (conduction) band edges, which can also create shallow charge trap (tail) states. There is, therefore, the intriguing possibility that residual microstrain is a significant cause of charge-carrier trapping in organic semiconductors. Proving this would be a major conceptual step in clarifying the link between structural and electronic disorder, a long-sought goal for organic electronics. However, a challenge is that, as far as we are aware, the quantitative connection between electronic structure and mechanical strain has never been established in these materials.

Here we take a considerable step in this direction by measuring the effect of tensile and compressive strains on the work function (WF) of a prototypical p-type organic semiconductor for the first time. The WF is defined as the energy difference between the vacuum level (*E*_vac_) and the Fermi level (*E*_F_), that is, WF=*E*_vac_−*E*_F_. Strain can modify *E*_vac_ and *E*_F_ of a semiconductor by changing the band-edge positions, the dopant levels in the band gap or the surface dipoles. Thus, strain impacts the WF. In a p-type crystal of *π*-conjugated organic molecules in which the Fermi level is pinned (fixed doping level), strain effects on *E*_F_ can be pictured, to a first approximation, as follows: compressive strains increase the frontier orbital (for example, HOMO) overlap of adjacent molecules, leading to a wider valence band^24^, a higher *E*_F_, and a lower WF. Conversely, tensile strains increase the separation between molecules, lowering orbital overlap and decreasing valence bandwidth and *E*_F_, thus increasing the WF. Note that with fixed doping level, the WF change can be directly translated to the change of the ionization potential (IP=*E*_vac_−*E*_HOMO_), which is a more commonly adopted term for describing the electronic structure of intrinsic organic semiconductors.

Our measurements focus on p-type rubrene single crystals, which serve as a model material platform for many fundamental studies of organic semiconductor physics due to their exemplary transport properties, that is, the highest reproducible charge-carrier mobilities to date have been achieved in single crystal rubrene field-effect transistors^26^. By adhering thin rubrene crystals onto substrates with coefficients of thermal expansion (CTEs) distinctly different from rubrene and varying the temperature, we systematically induce large and controlled tensile or compressive strains in rubrene crystals and quantify the elastic portion by X-ray diffraction. The corresponding WF of rubrene, measured by temperature-dependent scanning Kelvin probe microscopy (SKPM), is found to increase (decrease) with the in-plane tensile (compressive) strain. The measured changes in WF (ΔWF) are qualitatively confirmed by density functional theory (DFT) calculations, and verify that indeed small elastic strains <0.1% can lead to ΔWF surpassing the room temperature thermal energy *k*T=25 meV. Furthermore, we find that the onset of tensile plastic strain leads to even larger increases of WF with strain. These findings constitute a definitive link between structural deformation and electronic disorder in a model organic semiconductor.

## Results

### Rubrene single crystals

**Figure 1:**
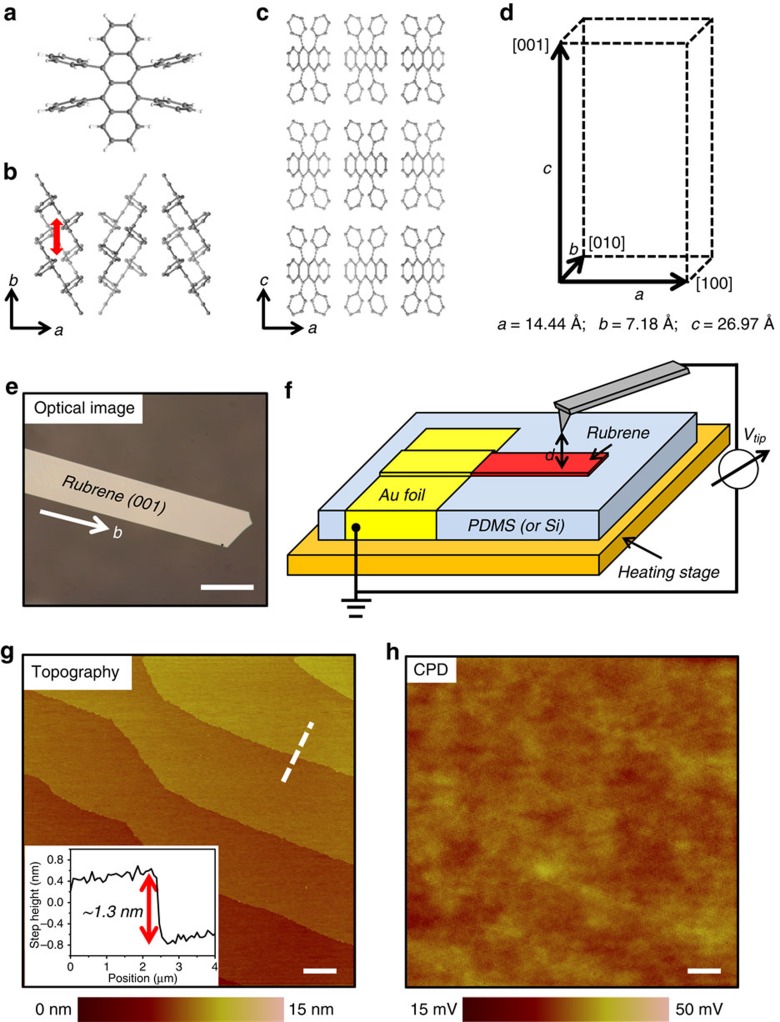
Crystal structure and SKPM measurement of rubrene single crystals. (**a**) Molecular structure of rubrene. (**b**) Crystal structure in the *a-b* plane; red arrow indicates the *π*-stacking interaction. (**c**) Crystal structure in the *a-c* plane. (**d**) Orthorhombic structure and lattice parameters of rubrene. (**e**) Optical micrograph of as-grown rubrene crystal. Length of scale bar, 200 μm. (**f**) SKPM setup for CPD measurement. The sample sits on top of a heating stage and is grounded through gold foil. The conducting probe scans in a two-pass ‘lift mode' and a constant lift height (*d*=50 nm) is used in the ‘interleave' pass. (**g**) Topography of rubrene single crystal shows typical terrace structure and each terrace has height corresponding to one molecular layer. Length of scale bar, 2 μm. Inset: step height profile of the dashed line. (**h**) CPD image obtained simultaneously with topography shows nearly homogeneous CPD across the surface. Length of scale bar is 2 μm.

The crystal structure of rubrene single crystals grown from the vapour phase is shown in Fig. 1a–d. The crystals adopt an orthorhombic structure and slipped *π*-stack packing motif with the *π*-stacking direction along the *b* axis^27^. The optical micrograph (Fig. 1e) shows a lath-like single crystal with the largest facet being the (001) plane and the longest direction aligned with the *b* axis. Thin rubrene crystals (∼2 μm) are then laminated on poly(dimethylsiloxane) (PDMS) or silicon (Si) substrates for X-ray diffraction and SKPM measurements. PDMS is chosen for its significantly larger CTE (∼300 × 10^−6^ K^−1^) than that of rubrene (∼10–80 × 10^−6^ K^−1^)^28^,^29^ in order to induce tensile strain in laminated rubrene crystals upon increasing the temperature. Si, on the other hand, is chosen to induce compressive strain since it has a much smaller CTE (3–4 × 10^–6^ K^−1^) than that of rubrene (Supplementary Table 1).

Figure 1f shows the scheme of the SKPM measurement, which operates in a two-pass ‘lift mode' to acquire images of topography and the contact potential difference (CPD) between the tip and the sample^30^. The CPD is related to the WF of the tip and the sample by *q*CPD=WF_tip_−WF_sample_, where *q* is the elementary charge. Therefore, with the same tip acting as the reference, the measured CPD change reflects the WF change of the sample, that is, *q*ΔCPD=−ΔWF_sample_=−ΔWF^31^,^32^. Typical topography and CPD images of a rubrene (001) surface obtained by SKPM at room temperature are shown in Fig. 1g,h, respectively. The topography image shows a surface with several molecularly flat terraces, which extend over a distance of several micrometres. The height of each terrace obtained from the profile of the white dashed line, as shown in the inset of Fig. 1g, is ∼13 Å, which coincides with one-half of the *c* axis unit cell parameter. This is in agreement with previous observations of rubrene molecular steps and confirms that there are two nonequivalent molecular planes in the rubrene crystal unit cell^33^. The CPD map, on the other hand, is almost featureless. The local CPD variations, characterized by the root-mean-square CPD roughness, are less than 3 mV for this 20 × 20 μm^2^ area. The CPD map thus suggests that the rubrene (001) surface is electrostatically homogeneous, in accordance with the high structural order of rubrene single crystals. It also demonstrates the high voltage resolution of interleave-based SKPM (about 1 mV), which allows small WF changes of the sample to be resolved.

### Strain quantification by *in situ* X-ray diffraction studies

**Figure 2:**
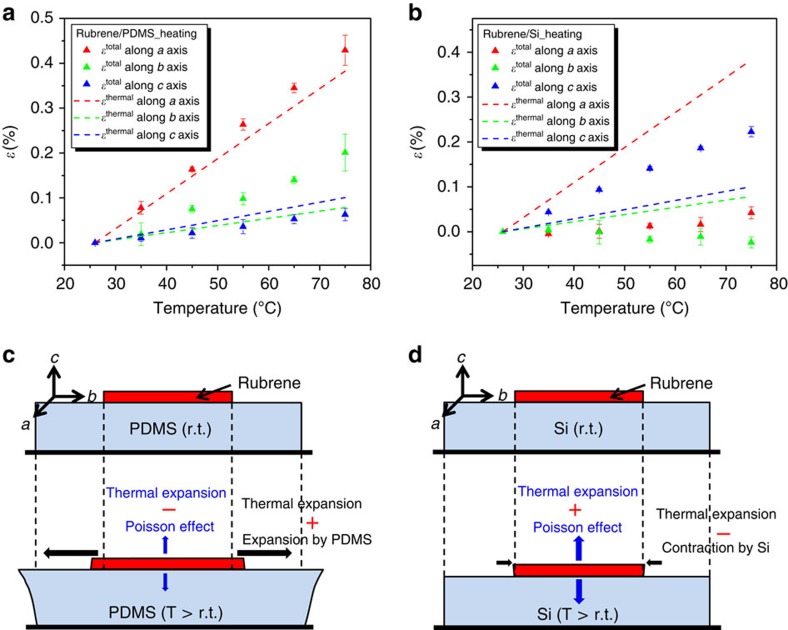
Total elastic strain in rubrene single crystals. (**a**) Average total elastic strains *ɛ*^total^ of rubrene along *a*, *b* and *c* axes as a function of temperature for crystals laminated on PDMS, which are different from the corresponding thermal strains *ɛ*^thermal^ expected for free rubrene crystals (dashed lines). (**b**) Average *ɛ*^total^ of rubrene along *a*, *b* and *c* axes as a function of temperature for crystals laminated on Si, different from the corresponding *ɛ*^thermal^ predicted for free rubrene crystals (dashed lines). The error bars in *ɛ*^total^ were based on the s.d. calculated as demonstrated in Supplementary Table 2. (**c**) Illustration of strain components for rubrene on PDMS. (**d**) Illustration of strain components for rubrene on Si.

To quantify the elastic strain induced in rubrene crystals, temperature-dependent X-ray diffraction measurements were carried out. By recording the 2*θ* shifts of rubrene (0012), (113) and (313) peaks as a function of temperature and calculating the corresponding d-spacings, *d*_0012_, *d*_113_ and *d*_313_, the total elastic strains (*ɛ*^total^) along the rubrene *a*, *b* and *c* axes at different temperatures were computed (Supplementary Methods, Supplementary Figs 1-3 and Supplementary Table 2), which are composed of both induced mechanical strain (*ɛ*^elastic^) and thermal strain (*ɛ*^thermal^), that is, *ɛ*^total^=*ɛ*^elastic^+*ɛ*^thermal^. Figures 2a,b show the average *ɛ*^*total*^ for rubrene on PDMS and rubrene on Si, respectively, when the samples were heated from room temperature to 75 °C. Also shown are the calculated thermal strains *ɛ*^thermal^ for free crystals using the reported CTEs (Supplementary Table 1)^28^. Clearly, the two types of samples show distinct strain behaviours with increasing temperature. In Fig. 2a, rubrene crystals laminated on PDMS exhibit slightly larger *ɛ*^total^ along the *a* axis and much larger *ɛ*^total^ along the *b* axis compared with *ɛ*^thermal^ estimated for free crystals. *ɛ*^total^ along the *c* axis of rubrene on PDMS, however, is slightly smaller than the estimated *ɛ*^thermal^ for free crystals. On the other hand, crystals on Si show essentially the opposite behaviour, namely much smaller *ɛ*^total^ in the *a–b* plane and much larger *ɛ*^total^ along the *c* axis than the corresponding *ɛ*^thermal^ predicted for free rubrene.

Such different strain states for rubrene on PDMS and rubrene on Si are explained by the schematics in Fig. 2c,d. For rubrene on PDMS, significant expansion of PDMS due to its large CTE induces tensile strains in the rubrene *a–b* plane, resulting in *ɛ*^total^ greater than the estimated *ɛ*^thermal^ of free crystals. The difference in *ɛ*^total^ for the *a* and *b* axes with respect to the corresponding *ɛ*^thermal^ may be attributed to the anisotropic CTE of rubrene^28^,^29^, that is, CTE along the *a* axis (∼78 × 10^−6^ K^−1^) is significantly larger than that along the *b* axis (∼16 × 10^−6^ K^−1^), which leads to anisotropic CTE mismatch between the crystal and PDMS. Furthermore, as a result of the Poisson effect, the substrate-induced in-plane tensile strain in rubrene exerts a compressive component in the out-of-plane direction, or the *c* axis. Therefore, *ɛ*^total^ measured in the *c* axis is smaller than the predicted *ɛ*^thermal^ if the crystal only undergoes thermal expansion.

In contrast, the Si substrate has negligible expansion (∼0.015%) within the temperature range of interest such that the thermal expansion in the *a*–*b* plane of rubrene is largely constrained. The *a* and *b* axes experience compression induced by the substrate, offsetting thermal expansion and thus leading to very small *ɛ*^total^. Similarly, due to the Poisson effect, the compressive strain creates tension in the *c* axis, making *ɛ*^total^ along the *c* axis much larger than the *c* axis thermal expansion expected for free crystals.

**Figure 3:**
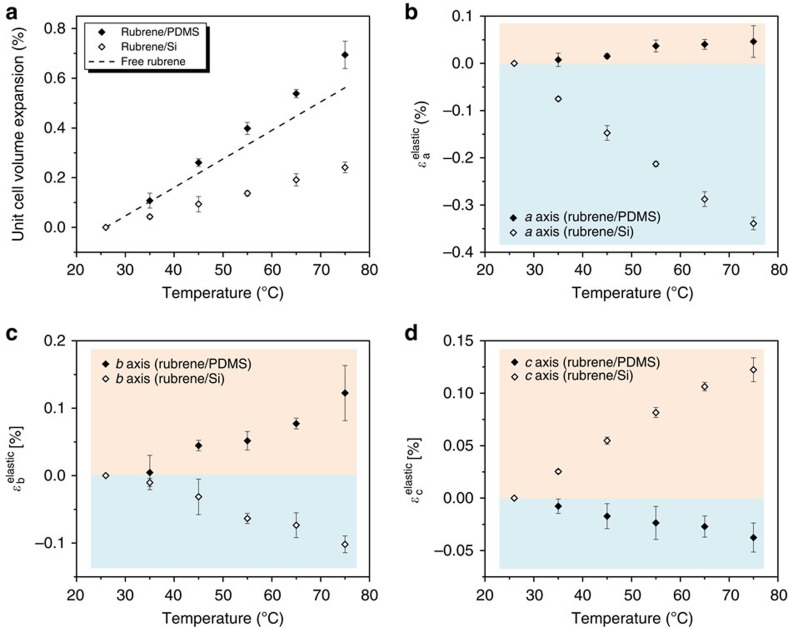
Unit cell volume expansion and substrate-induced elastic mechanical strain. (**a**) The unit cell volume expansion for rubrene crystals laminated on PDMS and Si as a function of temperature, which are compared with that expected for free crystals. The error bars in unit cell volume expansion were based on the propagation of s.d. errors in *ɛ*^total^ of the three axes as shown in Fig. 2a,b. (**b**) Average induced mechanical strain (*ɛ*^elastic^=*ɛ*^total^−*ɛ*^thermal^) along rubrene *a* axis as a function of temperature for crystals laminated on PDMS and Si, respectively. (**c**) Average *ɛ*^elastic^ along rubrene *b* axis as a function of temperature for crystals laminated on PDMS and Si, respectively. (**d**) Average *ɛ*^elastic^ along rubrene *c* axis as a function of temperature for crystals laminated on PDMS and Si, respectively. The error bars in *ɛ*^elastic^ were based on the standard deviation errors in *ɛ*^total^ as shown in Fig. 2a,b.

The overall tension and compression states of rubrene crystals laminated on PDMS and Si, respectively, are illustrated quantitatively by the percentages of unit cell volume expansion as shown in Fig. 3a. Rubrene on PDMS exhibits a total volume increase of 0.7%, slightly larger than that estimated for free crystals, consistent with net tension, whereas the total volume increases by only 0.25% for rubrene on Si, much smaller compared with the computed free crystal result and consistent with net compression. Though the CTE mismatch between rubrene and PDMS is greater, the magnitude of the tensile strain induced in rubrene by PDMS is actually smaller than the compressive strain induced by Si. This can be understood by the relative stiffness of rubrene compared with the substrates. Rubrene with modulus of ∼15 GPa is ∼10 times softer than Si whereas it is over 10^4^ times stiffer than PDMS^34^. The complexity introduced by the compounding effects of CTE mismatch, relative stiffness and interface adhesion is such that the elastic state of the rubrene crystal cannot be calculated and must be accessed experimentally by *in situ* X-ray diffraction measurements.

The net elastic mechanical strains (*ɛ*^elastic^*=ɛ*^total^*−ɛ*^thermal^) along the three principal axes of rubrene on PDMS and Si are shown in Fig. 3b−d. For rubrene on PDMS, tensile strain reaches ∼0.05% along the *a* axis and over 0.1% along the *b* axis; the *c* axis compressive strain reaches approximately −0.04%. For rubrene on Si, the *a* and *b* axes show large compressive strain, approximately −0.35% and −0.1%, respectively; the tensile strain along the *c* axis reaches over 0.1%. We will show below that the differences in *ɛ*^elastic^ for the two types of samples correlate with dramatically different WF changes.

### WF measurements by temperature-dependent SKPM

**Figure 4:**
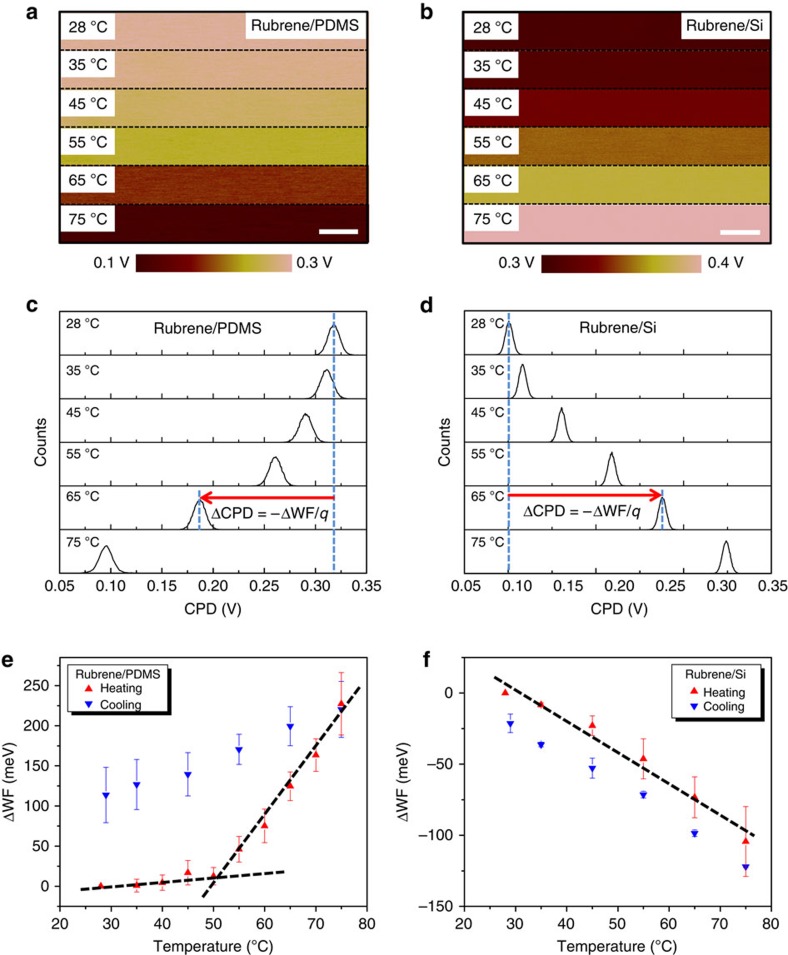
SKPM measurements of rubrene as a function of temperature. (**a**) CPD of rubrene on PDMS shifts from more positive (bright) to more negative (dark) when the sample is heated from room temperature to 75 °C. Length of scale bar is 500 nm. (**b**) CPD of rubrene on Si shifts from more negative (dark) to more positive (bright) when the sample is heated from room temperature to 75 °C. Length of scale bar, 500 nm. (**c**) CPD histograms extracted from images in **a**. ΔCPD, indicated by the red arrow, is defined as the change of CPD at any elevated temperature from that at room temperature, that is, ΔCPD=CPD(T)−CPD(r.t.). (**d**) CPD histograms extracted from images in **b**. (**e**) Average ΔWF of rubrene on PDMS as a function of temperature with ΔWF defined as −*q*ΔCPD, where *q* is the elementary charge. The dashed lines show two distinct regimes of ΔWF at low and high temperatures. The error bars in ΔWF were based on the s.d. of 24 independent measurements from four different samples at each temperature. (**f**) Average ΔWF of rubrene on Si as a function of temperature. The dashed line shows continuous change of ΔWF within the entire temperature range. The error bars in ΔWF were based on s.d. of 12 independent measurements from two different samples at each temperature.

To measure the WF change of rubrene as a function of its mechanical states, SKPM as described above was carried out at different temperatures with the same tip. Note that the WF change of the Pt-coated tip with temperature is not significant over the temperature range of interest because the WFs of metals typically exhibit a weak temperature dependence (∼10^−4^ eV K^−1^)^35^. Figure 4a,b show the CPD maps of rubrene crystals on PDMS and Si from room temperature to 75 °C. As temperature increases, the CPD of the rubrene (001) surface decreases (bright to dark) on PDMS and increases (dark to bright) on Si. The CPD evolutions are illustrated quantitatively with the histograms extracted from the CPD maps at different temperatures, as shown in Fig. 4c,d. For rubrene on PDMS (Fig. 4c), the CPD changes by more than −200 mV from room temperature to 75 °C, whereas for rubrene on Si (Fig. 4d), it changes by about +120 mV across the same temperature range.

By averaging multiple measurements from multiple samples, the average change in WF (ΔWF=−*q*ΔCPD) as a function of temperature is plotted for rubrene on PDMS (Fig. 4e) and rubrene on Si (Fig. 4f), respectively, for samples undergoing consecutive heating and cooling cycles. As shown in Fig. 4e, the WF of rubrene increases (ΔWF>0) with increasing temperature on PDMS and there is an evident transition when the temperature is close to 50 °C. First, the WF increases only slightly from room temperature to ∼50 °C and then above 50 °C it increases much more steeply. As will be discussed later, we attribute the slope change to the elastic-to-plastic transition. Upon cooling, the WF of rubrene decreases continuously (ΔWF<0) and there remains a large hysteresis of ∼100 meV on returning to room temperature. An opposite trend is observed in rubrene crystals on Si, as shown in Fig. 4f. The WF of rubrene decreases (ΔWF<0) with increasing temperature, and unlike rubrene on PDMS, there is no obvious slope change upon heating and the hysteresis between heating and cooling is almost negligible: the WF comes back to its original value at the end of the cycle. Overall, Fig. 4 demonstrates that changes in WF are significant for both types of samples and there is a qualitative difference in behaviour for rubrene on PDMS and rubrene on Si.

### Strain-WF Relationship and DFT calculations

**Figure 5:**
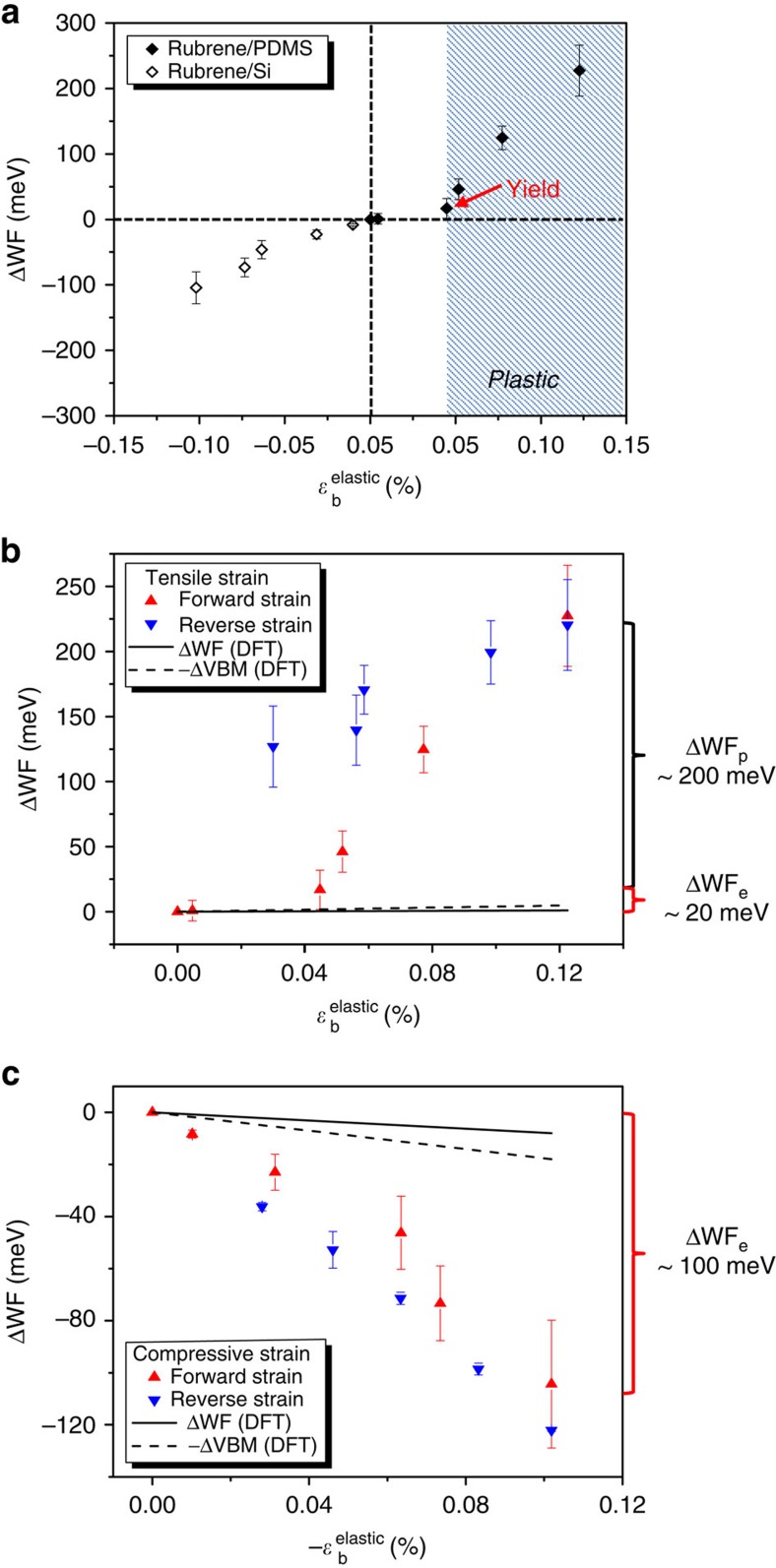
ΔWF as a function of substrate-induced elastic strain. (**a**) ΔWF as a function of *ɛ*^elastic^ (tensile and compressive strain) along the *b* axis. The WF increases with tensile strain and decreases with compressive strain. Note that ΔWF in the shaded region is mainly a result of plastic deformation instead of elastic tensile strain. (**b**) ΔWF as a function of elastic tensile strain for forward and reverse strains. There is an elastic-to-plastic transition characterized by a sudden rise in ΔWF with strain. There is a large hysteresis of ΔWF between the forward and reverse tensile strains, indicating the effect of plastic deformation. The DFT calculation results are shown by the dashed line (−ΔVBM) and the solid line (ΔWF). The calculated −ΔVBM and ΔWF slightly increase with elastic tensile strain, a trend that qualitatively agrees with the experimental results. The quantitative disagreement between the calculations and the experimental results in the high-strain regime is attributed to plastic deformation, which is not considered in the calculations. (**c**) ΔWF as a function of elastic compressive strain for forward and reverse strains. The WF decreases with strain smoothly and no apparent elastic-to-plastic transition is observed. There is negligible hysteresis of ΔWF between the forward and reverse strains. The DFT calculation results are shown by the dashed line (−ΔVBM) and the solid line (ΔWF). The calculated −ΔVBM and ΔWF decrease with elastic compressive strain, a trend that qualitatively agrees with the experimental results. The deviation in the ΔWF magnitude of the calculations from the experiments may be due to dynamic effects that are not considered in the theoretical approach. The errors bars in ΔWF were based on standard deviation errors as described in Fig. 4.

Figure 5 illustrates ΔWF as a function of *ɛ*^elastic^ (tensile and compressive) in rubrene. ΔWF is plotted versus the *b* axis elastic strain for simplicity because it is reasonable to suppose that the relative significance of mechanical strains along different axes is positively correlated with the strength of intermolecular interactions, that is, *b* axis (*π*-stacking direction)>*a* axis>>*c* axis. It is evident from Fig. 5a that WF increases significantly with tensile strain and decreases with compressive strain, and that the changes can be much greater than *k*T (25 meV) at room temperature. A similar trend of the WF change has been observed or predicted in strained Si and graphene^36^,^37^,^38^,^39^. Figures 5b,c show ΔWF as a function of forward and reverse elastic strains on PDMS and Si substrates, respectively.

We attribute the measured ΔWFs to the substrate-induced mechanical strains *ɛ*^elastic^ in rubrene crystals—instead of intrinsic changes with temperature or surface contamination—for several reasons. First, ΔWF is completely opposite for rubrene on PDMS versus rubrene on Si even though they experience the same temperature change. This rules out temperature as the dominating factor for ΔWF. Second, although in general the WF may be affected by surface contamination, it is unlikely to result in the systematic WF trends we observed, including the hysteresis in rubrene on PDMS. Third, we performed static level (*T*=0 K) DFT calculations of ΔWF as a function of strain as is shown in Fig. 5b,c. Although they do not match quantitatively, the calculations predict the same signs for ΔWF as observed experimentally and thus qualitatively support our conclusion that ΔWF is dominated by mechanical strain (Supplementary Table 3).

To estimate ΔWF by DFT, the evolution of the valence band maximum (VBM) and the potential energy of an electron at the vacuum level (*E*_vac_) were calculated based on the crystal structures of rubrene under the experimentally-attained mechanical strains; we set WF=*E*_vac_−*E*_F_≈*E*_vac_−VBM such that ΔWF≈Δ*E*_vac_−ΔVBM. This definition of WF is justified because we expect *E*_F_ of rubrene to lie closer to the VBM and to track the VBM because of Fermi level pinning or partial pinning, that is, the offset between *E*_F_ and VBM is set by the natural p-type doping of as-grown rubrene. From Fig. 5b,c, it is clear that the signs of the calculated ΔWF are determined by ΔVBM. Further, the calculated ΔVBM is consistent with qualitative expectations, namely that tensile strain decreases HOMO–HOMO (*π*–*π*) interaction, lowering the VBM and thus increasing the WF (ΔWF>0). Compressive strain, on the other hand, increases the *π*–*π* interaction, which increases the VBM and decreases the WF (ΔWF<0).

The discrepancy between the calculations and experiments may have several origins. First, we note that the calculations did not and cannot take into account plastic deformation that occurs for the case of rubrene on PDMS. So in this case, comparison is only fair at the lowest strains, and here the number of data points is sparse. Second, within the elastic strain regime, the calculations only simulate a static picture, whereas dynamic effects with increasing temperature (for example, local/non-local electron–phonon couplings) are not considered. Such dynamic effects are expected to be more significant in the case of compressive strain. Indeed, a larger quantitative discrepancy with the experimental results is observed for compressive elastic strain (Fig. 5c).

The WF trend in the high-tension regime (Fig. 5a,b) is ascribed primarily to the effect of plastic deformation based on two experimental observations. One is the sudden steep rise in ΔWF at ∼0.05% strain, which is not associated with any noticeable signs of surface roughening or structural instability (for example, cracks). It is known from the literature that yield strains for organic crystals are typically <0.1% (ref. 40), consistent with our assignment. A similar WF-strain relationship on yielding has also been reported for metals^41^. The other important observation is the large hysteresis in ΔWF when elastic tensile strain recovers. This non-recoverable ΔWF is also strongly indicative of plastic strain. In compression, however, ΔWF decreases smoothly as a function of strain without any significant transition point or hysteresis, suggesting that the yield point is not reached. The yield strain is therefore higher for compressive strain than tensile strain, which is also in agreement with observations in other materials and could be due to larger friction between slip layers under compression^41^. The association of plastic deformation with a large non-recoverable ΔWF strongly suggests the existence of a relationship between defects such as dislocations and charge trapping in organic films.

## Discussion

Controlled and quantifiable tensile and compressive strains have been induced in rubrene single crystals by utilizing thermal expansion of the substrates. The change of WF as a result of induced strain is measured by SKPM. The WF increases with tensile strain (elastic and plastic) and decreases with compressive strain in the *a–b* plane of rubrene crystals, confirmed qualitatively by DFT calculations. In tension, the WF increase is slight but measurable by SKPM in the elastic regime. The WF increase becomes much more significant upon the onset of plastic deformation, which occurs at a relatively small tensile strain (<0.1%). In compression, the WF decreases smoothly with increasing strain and no apparent cross-over from elastic to plastic behaviour is observed within the investigated strain range. We propose that strain-induced WF variations will lead to band-edge fluctuations, which can impact charge transport, and are an important mechanism for the creation of electrostatic disorder (for example, band-tail states) in organic semiconductors. As organic thin films will typically have residual microstrain, the sensitivity of WF to strain in organic semiconductors has great implications for the fundamental electrical properties of these materials and their performance in devices.

## Methods

### Sample preparation

Rubrene single crystals were grown by physical vapour transport using ultrapure Ar as carrier gas^42^. Commercially available rubrene (sublimed grade, 99.99%, Sigma-Aldrich) was used as received as the source material. The sublimation temperature was ∼280 °C. Only thin crystals (<5 μm) with uniform crystalline regions and smooth/clean surfaces were selected for sample preparation. Thicknesses of the crystals were measured by surface profilometry (KLA-Tencor P-16 surface profiler). The average thickness of all crystals used was ∼2 μm. Freshly made crystals were laminated onto either PDMS or silicon substrates. Spontaneous adhesion of the crystals to the substrates occurred. In order to electrically ground the sample, vapour deposited gold film with thicknesses around 500 nm was removed from a Si substrate and transferred to cover part of the crystal by tweezers, and silver paint was then used to connect the gold film to the metal SKPM sample puck.

### Scanning Kelvin probe microscopy

SKPM measurements were performed with a Cypher ES Environmental AFM (Asylum Research), which works in a two pass ‘lift mode'. In the first pass of each line of an image, the conductive probe scans the rubrene surface in attractive-regime dynamic mode to generate the topographic data under conventional amplitude-modulation feedback (also known as ‘AC' or ‘tapping' mode) while mechanically vibrating the cantilever near resonance. The attractive-regime dynamic mode was used since it better preserves the probe and thereby allows meaningful CPD comparison among different measurements^32^. To stabilize performance in the attractive regime, the cantilever was driven at a drive frequency slightly larger (∼150 Hz) than the fundamental resonant frequency, and the setpoint amplitude was about 90% of the free amplitude (∼90 nm). In the second ‘interleave' pass, the probe was lifted to a constant height above the surface and scanned the topographic trajectory acquired in the first pass. A tip-applied AC bias resonantly excited the cantilever (via a time-varying electrostatic force gradient between tip and sample) while a DC bias was adjusted under feedback so as to null the AC excitation by matching (and thus measuring) the CPD point-to-point across the surface^30^. The samples were heated with an integrated heating stage at a heating rate of ∼2 °C min^−1^. The samples were held for ∼10 min at the target temperature and the CPDs of the same area were measured multiple times. Note that the cantilever vibrational tuning was repeated at each temperature to account for changes in resonant frequency with temperature. The typical probes were from Nanosensors (PPP-EFM-W, Pt/Ir coated, resonant frequency ∼75 kHz, spring constant ∼2.8 N m^−1^). The lift height during the second pass was 50 nm, which was beyond the range where van der Waals forces come into play. The applied AC voltage in SKPM was 0±3 V in amplitude. The SKPM images were analysed using the freeware Gwyddion.

### X-ray diffraction

*In situ* X-ray diffraction measurements were carried out with a PANalytical X'Pert Pro X-ray diffractometer with a Cu K*α* source operated at 45 kV and 40 mA filament current. The samples were slowly heated (∼2 °C min^−1^) to target temperatures using a thermal stage connected to an Anton Paar temperature control unit (TCU 150) within the diffractometer. The samples were allowed to stabilize at the target temperature for ∼10 min before the diffraction peaks of interest were measured. To more accurately study the weakly diffracting off-axis peaks, two-dimensional reciprocal space maps were measured. Detailed calculations of strain based X-ray diffraction measurements are shown in Supplementary Methods, Supplementary Figs 1–3 and Supplementary Table 2.

### DFT calculations

The geometric and electronic properties of strained rubrene single crystals were computed at the DFT level with the VASP code^43^. The Perdew-Burke-Ernzerhof (PBE) functional were used with a plane-wave basis set (300 eV cutoff) and projector augmented wave potentials^44^,^45^. The DFT calculations were carried out using on a 2 × 2 × 1 *k*-point grid and a Gaussian smearing with 0.10 eV width. The calculations considered the effects due to elastic strain in a static sense (that is, the dynamic motions of the molecules at a given temperature were not explicitly considered), with the unit-cell parameters chosen as those modified by mechanical strain obtained experimentally. For each crystal structure, the molecules within a unit cell were allowed to fully relax while the lattice parameters remained fixed to the experimental values. Next, a periodic slab containing two layers of rubrene molecules was extracted from the three-dimensional structure, with 30 Å of vacuum space placed between each slab (to prevent spurious inter-slab interactions). The molecules were then allowed to further relax within the fixed surface (slab) unit cell. The WF was determined by tracking the evolution of the potential energy of an electron at the vacuum level (*E*_vac_) and the VBM, as a function of the change in the (experimentally observed) lattice parameters due to mechanical strain.

## Additional information

**How to cite this article:** Wu. Y. *et al.* Strain effects on the work function of an organic semiconductor. *Nat. Commun.* 7:10270 doi: 10.1038/ncomms10270 (2016).

## Supplementary Material

Supplementary Information Supplementary Figures 1-3, Supplementary Tables 1-3, Supplementary Methods and Supplementary References
