# Supplementary material for: Strain effects on the work function of an organic semiconductor
Source: Nat Commun. 2016 Feb 1;7:10270. doi: 10.1038/ncomms10270 (PMC4740348; doi:10.1038/ncomms10270)
Supplement: Supplementary Information — Supplementary Figures 1-3, Supplementary Tables 1-3, Supplementary Methods and Supplementary References [file ncomms10270-s1.pdf]

Supplementary Information

Supplementary Figures

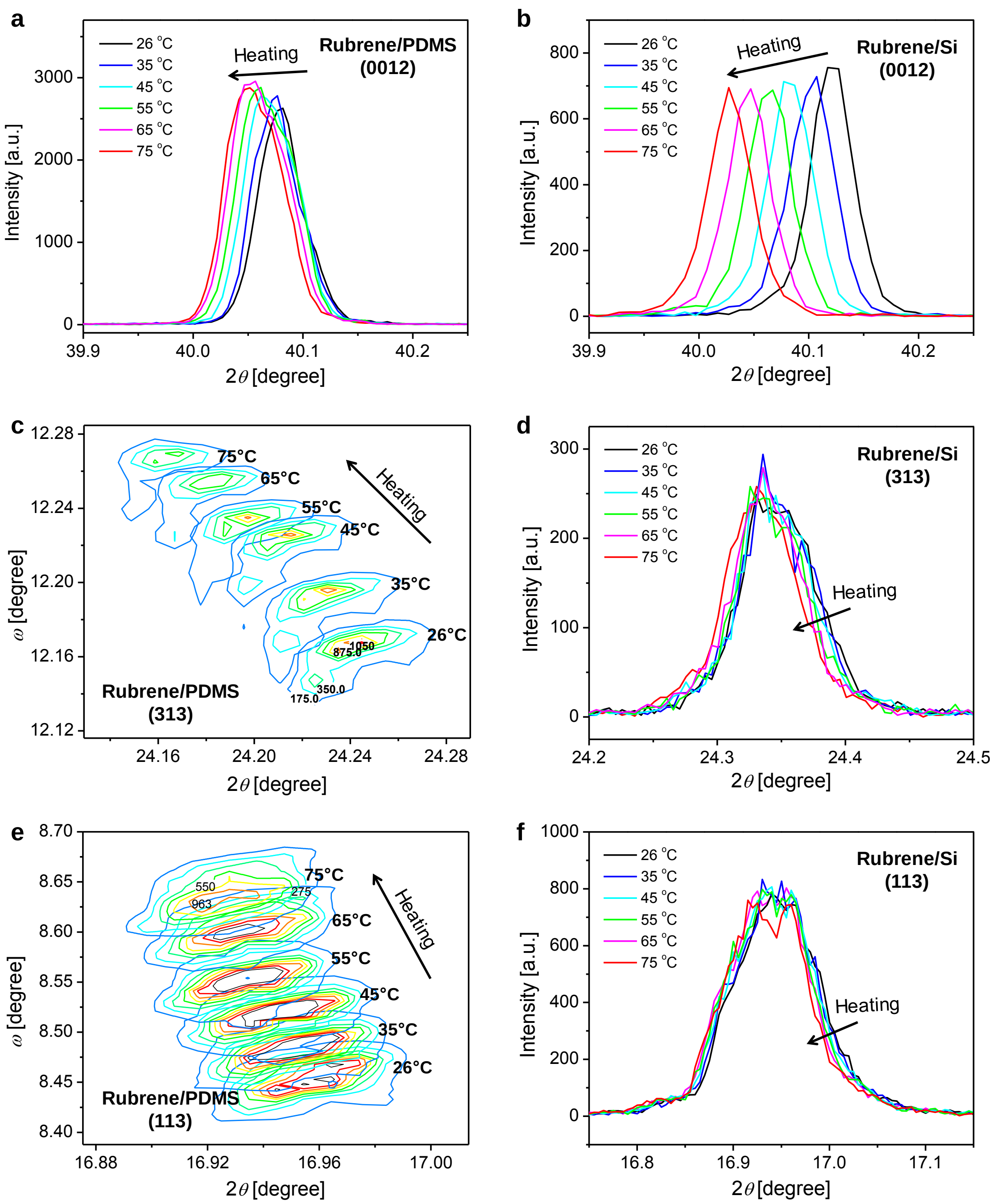

Supplementary Figure 1 | XRD measurements of 2*θ* as a function of temperature for the (0012), (313), and (113) diffraction peaks of rubrene laminated on PDMS and Si.

(**a**) Out-of-plane 2*θ*-*ω* coupled scan of rubrene (0012) diffraction peak upon heating for rubrene on PDMS. (**b**) Out-of-plane 2*θ*-*ω* coupled scan of rubrene (0012) diffraction peak upon heating for rubrene on Si. (**c**) 2D reciprocal space mapping of rubrene (313) diffraction peak upon heating for rubrene on PDMS. 2D reciprocal space mapping instead of 2*θ*-*ω* coupled scan was used since the (313) diffraction peak for rubrene on PDMS obtained by 2*θ*-*ω* coupled scan is very weak. (**d**) Off-axis 2*θ*-*ω* coupled scan of rubrene (313) diffraction peak upon heating for rubrene on Si. (**e**) 2D reciprocal space mapping of rubrene (113) diffraction peak upon heating for rubrene on PDMS. 2D reciprocal space mapping instead of 2*θ*-*ω* coupled scan was used since the (113) diffraction peak for rubrene on PDMS obtained by 2*θ*-*ω* coupled scan is very weak. (**f**) Off-axis 2*θ*-*ω* coupled scan of rubrene (113) diffraction peak upon heating for rubrene on Si.

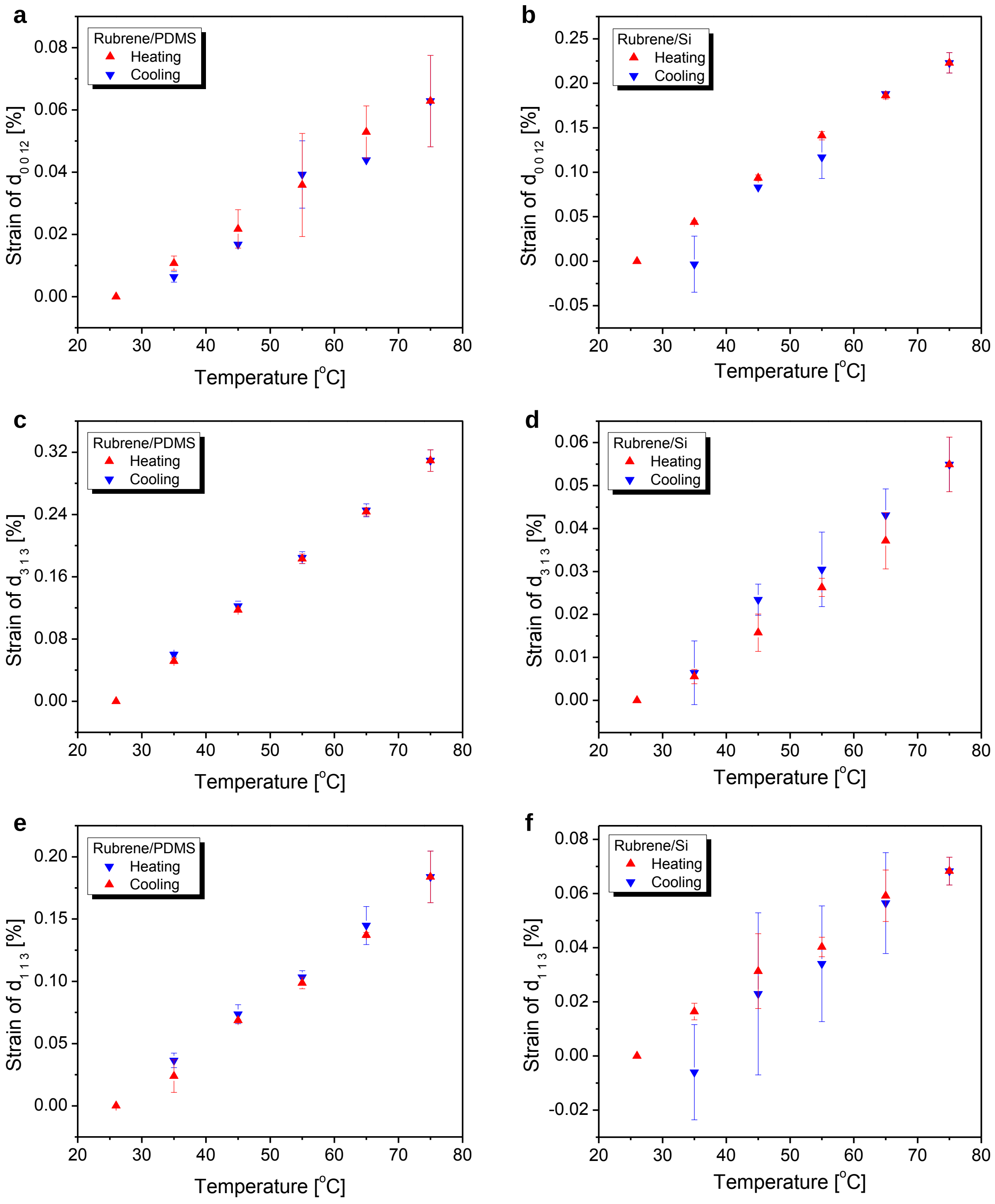

Supplementary Figure 2 | Average strains of the d-spacings, d_{0012}, d_{313}, and d_{113}, as a function of temperature for rubrene on PDMS and rubrene on Si.

(**a**) Average strain of d_{0012} as a function of temperature during heating and cooling obtained by measuring the (0012) peak shift of multiple rubrene samples on PDMS. (**b**) Average strain of

d_{0012} as a function of temperature during heating and cooling obtained by measuring the (0012) peak shift of multiple rubrene samples on Si. (**c**) Average strain of d_{313} as a function of temperature during heating and cooling obtained by measuring the (313) peak shift of multiple rubrene samples on PDMS. (**d**) Average strain of d_{313} as a function of temperature during heating and cooling obtained by measuring the (313) peak shift of multiple rubrene samples on Si. (**e**) Average strains of d_{113} as a function of temperature during heating and cooling obtained by measuring the (113) peak shift of multiple rubrene samples on PDMS. (**f**) Average strains of d_{113} as a function of temperature during heating and cooling obtained by measuring the (113) peak shift of multiple rubrene samples on Si. Errors bars in each d-spacing strains were based on standard deviations of 3-4 independent measurements.

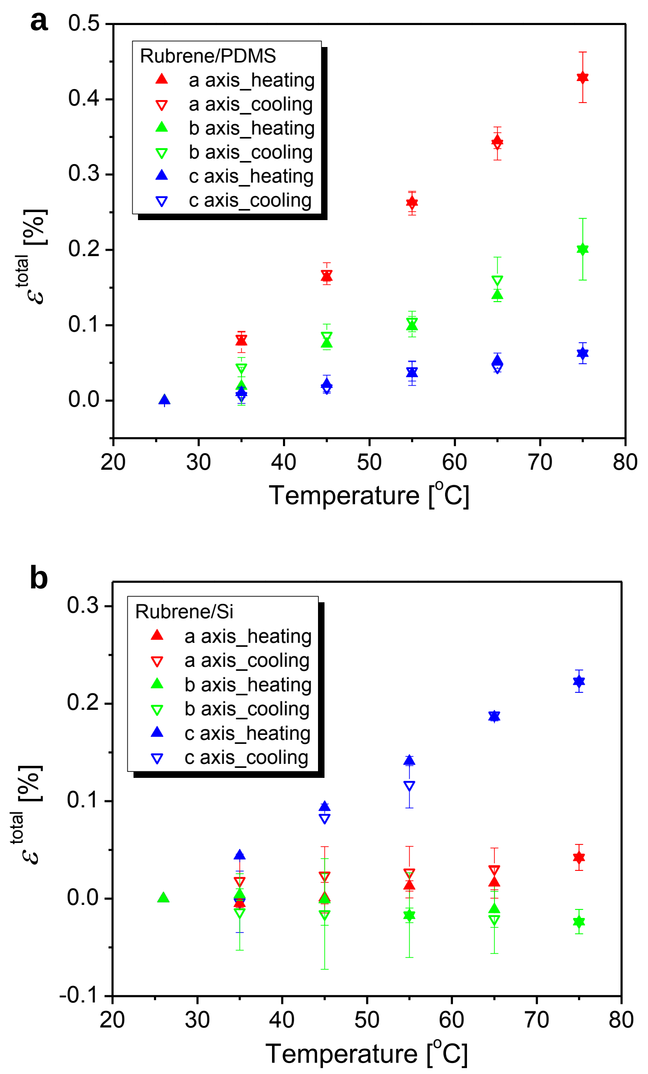

Supplementary Figure 3 | Reversibility of the average total elastic strain of rubrene on PDMS and rubrene on Si upon heating and cooling.

(**a**) Average total elastic strain ε^{total} of rubrene *a*, *b*, and *c* axes as a function of temperature for crystals on PDMS during a continuous heating and cooling cycle. (**b**) Average ε^{total} of rubrene *a*, *b*, and *c* axes as a function of temperature for crystals on Si during a continuous heating and cooling cycle. Both cases show very good reversibility of ε^{total}. Errors bars in ε^{total} were based on standard deviations calculated as illustrated in Supplementary Table 2.

Supplementary Tables

Supplementary Table 1. Coefficient of thermal expansion (CTE) of rubrene, PDMS, and Si.

**Materials**	**Rubrene**[1]			**PDMS**[2]	**Si**[3]
	a	***b***	***c***		
**CTE (10^{-6} K^{-1})**	78	16	20	300	3-4

Supplementary Table 2. Sample calculation of lattice parameters, lattice strains, and corresponding standard deviation errors based on measured average d-spacing strains of rubrene on PDMS at 55 °C.

Quantity	Unit	Value	Note
$\varepsilon\ d_{0012}$	%	0.03585	Average strain of d_{0012} for Rubrene/PDMS @ 55°C
$\delta\ (\varepsilon\ d_{0012})$	%	0.01581	Standard deviation of $\varepsilon\ d_{0012}$
$\varepsilon\ d_{313}$	%	0.18301	Average strain of d_{313} for Rubrene/PDMS @ 55°C
$\delta\ (\varepsilon\ d_{313})$	%	0.00608	Standard deviation of $\varepsilon\ d_{313}$
$\varepsilon\ d_{113}$	%	0.09868	Average strain of d_{113} for Rubrene/PDMS @ 55°C
$\delta\ (\varepsilon\ d_{113})$	%	0.00463	Standard deviation of $\varepsilon\ d_{113}$
d_{0012}	Å	2.248306	$d_{0012} = d_{0012}(r.t.) \times (1 + \varepsilon\ d_{0012})$; $d_{0012}(r.t.) = 2.2475$ Å
$\delta\ (d_{0012})$	Å	0.000355	$\delta\ (d_{0012}) = d_{0012}(r.t.) \times \delta\ (\varepsilon\ d_{0012})$
d_{313}	Å	3.659788	$d_{313} = d_{313}(r.t.) \times (1 + \varepsilon\ d_{313})$; $d_{313}(r.t.) = 3.653102$ Å
$\delta\ (d_{313})$	Å	0.000222	$\delta\ (d_{313}) = d_{313}(r.t.) \times \delta\ (\varepsilon\ d_{313})$
$1/(d_{313})^2$	Å^{-2}	0.07466	
$\delta\ (1/(d_{313})^2)$	Å^{-2}	9.0576×10^{-6}	$\delta\ (1/(d_{313})^2) = 2 \times \delta\ (d_{313})/(d_{313})^3$
d_{113}	Å	5.23462	$d_{113} = d_{113}(r.t.) \times (1 + \varepsilon\ d_{113})$; $d_{113}(r.t.) = 5.22946$ Å
$\delta\ (d_{113})$	Å	0.000242	$\delta\ (d_{113}) = d_{113}(r.t.) \times \delta\ (\varepsilon\ d_{113})$
$1/(d_{113})^2$	Å^{-2}	0.036495	
$\delta\ (1/(d_{113})^2)$	Å^{-2}	3.3743×10^{-6}	$\delta\ (1/(d_{113})^2) = 2 \times \delta\ (d_{113})/(\ d_{113})^3$
$\Delta 1$	Å^{-2}	0.038165	$\Delta 1 = 1/(d_{313})^2 - 1/(d_{113})^2$
$\delta\ (\Delta 1)$	Å^{-2}	9.6657×10^{-6}	$\delta\ (\Delta 1) = \{[\delta\ (1/(d_{313})^2)]^2 + [\delta\ (1/(d_{313})^2)]^2\}^{1/2}$
**c**	Å	26.97967	$c = 12 \times d_{0012}$
**δ (c)**	Å	0.00426	$\delta\ (c) = 12 \times \delta\ (d_{0012})$
**ε c**	%	0.03585	$\varepsilon\ c = \varepsilon\ d_{0012}$
**δ (ε c)**	%	0.01581	$\delta\ (\varepsilon\ c) = \delta\ (\varepsilon\ d_{0012})$
$9/c^2$	Å^{-2}	0.012364	
$\delta\ (9/c^2)$	Å^{-2}	3.9046×10^{-6}	$\delta\ (9/c^2) = 9 \times 2 \times \delta\ (c)/c^3$
**a**	Å	14.47813	$a = (8/\Delta 1)^{1/2}$
**δ (a)**	Å	0.001833	$\delta\ (a) = 0.5 \times a \times \delta\ (\Delta 1)/\Delta 1$
**ε a**	%	0.264058	$\varepsilon\ a = [a – a(r.t.)]/a(r.t.)$; $a(r.t.) = 14.44$ Å
**δ (ε a)**	%	0.012694	$\delta\ (\varepsilon\ a) = \delta\ (a)/a(r.t.)$
$1/a^2$	Å^{-2}	0.004771	
$\delta\ (1/a^2)$	Å^{-2}	1.208×10^{-6}	$\delta\ (1/a^2) = 2 \times \delta\ (a)/a^3$
$\Delta 2$	Å^{-2}	0.01936	$\Delta 2 = 1/(d_{113})^2 - 1/a^2 - 9/c^2$
$\delta\ (\Delta 2)$	Å^{-2}	5.3×10^{-6}	$\delta\ (\Delta 2) = \{[\delta\ (1/(d_{113})^2)]^2 + [\delta\ (1/a^2)]^2 + [\delta\ (9/c^2)]^2\}^{1/2}$
**b**	Å	7.18699	$b = (1/\Delta 2)^{1/2}$
**δ (b)**	Å	0.00098	$\delta\ (b) = 0.5 \times b \times \delta\ (\Delta 2)/\Delta 2$
**ε b**	%	0.09735	$\varepsilon\ b = [b – b(r.t.)]/b(r.t.)$; $b(r.t.) = 7.18$ Å
**δ (ε b)**	%	0.01365	$\delta\ (\varepsilon\ b) = \delta\ (b)/b(r.t.)$

Supplementary Table 3. Theoretical absolute values (in eV) of the potential energy at the vacuum level (E_{vac}), the valence band maximum (VBM), and the work function (WF) for rubrene on PDMS and rubrene on Si at different temperatures. The VBM values correspond to two-layer slabs, relaxed at fixed unit-cell parameters (taken from the experimental work). Each E_{vac} value is calculated at the vacuum level (30 Å thick vacuum layers added over the rubrene slabs). The electronic properties are calculated with the PBE functional.

	E_{vac} (eV)		ΔE_{vac} (eV)	VBM (eV)		ΔVBM (eV)	WF (eV)		ΔWF (eV)
T (°C)	26	75		26	75		26	75	
Rubrene/Si	2.463	2.473	0.010	-1.518	-1.500	0.018	3.981	3.973	-0.008
Rubrene/PDMS	2.463	2.459	-0.004	-1.518	-1.523	-0.005	3.981	3.982	0.001

Supplementary Methods

Strain measurements. XRD measurements were carried out to quantify the shift of 2θ positions of rubrene (0012), (313), and (113) diffraction peaks as a function of temperature. Examples of the raw data are shown in Supplementary Fig. 1. Out-of-plane 2θ-ω coupled scan was used to measure rubrene (0012) peak for both rubrene on PDMS and rubrene on Si. Off-axis 2θ-ω coupled scan was used to measure rubrene (313) and (113) diffraction peaks for rubrene on Si. The (313) and (113) peaks of rubrene on PDMS were measured by 2D reciprocal space mapping since these two peaks for rubrene on PDMS are very weak by off-axis 2θ-ω coupled scan and thus the accuracy is unsatisfactory. The corresponding d-spacings at different temperatures, d_{0012}, d_{313}, and d_{113}, can thus be determined with obtained 2θ by Bragg's law ($2d\sin\theta = n\lambda$). The changes of d-spacing at any elevated temperature relative to that at room temperature, i.e., d-spacing strains ($\varepsilon\ d_{hkl}$), were calculated for d_{0012}, d_{313}, and d_{113} and were averaged among multiple samples. The average d-spacing strains for rubrene on PDMS and rubrene on Si upon consecutive heating-cooling cycles are shown in Supplementary Fig. 2.

The average d-spacing strains along with their standard deviation errors were used to compute the total elastic strains along the three principal axes, a ($\varepsilon\ a$), b ($\varepsilon\ b$), and c ($\varepsilon\ c$) axes of rubrene. Since rubrene adopts an orthorhombic structure, the equation that relates the d-spacing d_{hkl} to the lattice parameters a, b, and c is given by

$$\frac{1}{{d_{hkl}}^2} = \frac{h^2}{a^2} + \frac{k^2}{b^2} + \frac{l^2}{c^2} \qquad \text{Supplementary Equation 1}$$

A sample calculation of *a*, *b*, *c*, *ε a*, *ε b*, *ε c*, as well as their standard deviation errors is shown in Supplementary Table 1. Supplementary Fig. 3 shows that the average total elastic strains (*ε a*, *ε b* and *ε c*) exhibit good reversibility upon heating and cooling.

Supplementary References

1. Jurchescu, O. D., Meetsma, A. & Palstra, T. T. M. Low-temperature structure of rubrene single crystals grown by vapor transport. *Acta Cryst.* **B62**, 330-334 (2006).

2. Bowden, N., et al. The controlled formation of ordered, sinusoidal structures by plasma oxidation of an elastomeric polymer. *Appl. Phys. Lett.* **75**, 2557-2559 (1999).

3. Okada, Y. & Tokumaru, Y. Precise determination of lattice parameter and thermal expansion coefficient of silicon between 300 and 1500 K. *J. Appl. Phys.* **56**, 314-320 (1984).
